# Commentary: Activation of Cortical Somatostatin Interneurons Rescues Synapse Loss and Motor Deficits After Acute MPTP Infusion

**DOI:** 10.3389/fncel.2019.00544

**Published:** 2019-12-17

**Authors:** Junhao Huang, Youguo Hao, Min Hu, Tifei Yuan

**Affiliations:** ^1^Guangdong Provincial Key Laboratory of Sports and Health Promotion, Scientific Research Center, Guangzhou Sport University, Guangzhou, China; ^2^Department of Rehabilitation, Shanghai Putuo People's Hospital, Shanghai, China; ^3^Shanghai Key Laboratory of Psychotic Disorders, Shanghai Mental Health Center, Shanghai Jiao Tong University School of Medicine, Shanghai, China; ^4^Co-innovation Center of Neuroregeneration, Nantong University, Nantong, China

**Keywords:** exercise, cortical inhibition, GABA, rehabilitation, movement disorder

Previous studies highlighted the destructive effects of neurotoxin (e.g., MPTP) on integrity of nigral dopamine system, accompanying series of changes in motor regulating network relate to striatal pathways. The fact that L-Dopa or deep brain stimulation (DBS) improves motor gaits in Parkinson's disease (PD) further strengthened the potential importance of subcortical circuits in motor ability control. A recent study highlighted the contribution of cortical inhibition in the process, and therefore might imply the cortical targeting strategy in motor disorder management (Chen et al., 2019a).

Chen et al. focused on synaptic integrity in motor cortex, and employed acute MPTP infusion model, which rapidly results in motor learning suppression. With two-photon based dendritic spine imaging, the authors quantified the turnover rate of pyramidal neuron spines at baseline and after motor learning, The results showed that MPTP exposure accelerated spine turnover (e.g., both formation and elimination), which might contribute to the decreased ability of motor learning. This rapid turnover correlates to enhanced apical dendrite Ca2+ spike generation, which is dependent on local NMDA receptors, the major source of calcium permeable glutamate receptors.

Somatostatin (SST) interneuron provides important sources to dendritic inhibition, while Parvalbumin (PV) preferably targets somatic region of cortical pyramidal neurons (Urban-Ciecko and Barth, 2016). The authors therefore examined the neuronal activity of SST interneurons in the motor cortex, and they detected significantly reduced calcium activity in SST interneurons, indicating the phenomenon of cortical dis-inhibition following MPTP exposure. Notably, with chemogenetic approach to re-activate SST interneuron, dendritic calcium spikes on pyramidal neurons return to baseline, together with a normal turnover rate for spikes, and motor learning ability, which is in a line with previous findings linking SST interneuron and motor learning (Adler et al., 2019).

The study validated the loss of cortical inhibition in acutely neurotoxic effect of MPTP, which further contributes to spine turnover changes and motor learning deficits. Very interestingly, reversing local inhibition at apical dendrite is sufficient to restore the spine dynamics and motor learning ability, which emphasized the therapeutic potential of targeting cortical inhibition in movement disorders (e.g., PD). Indeed, several studies reported aberrant neuronal activity at cortex and GABA deficits in PD patients or animal models of PD. In addition, L-dopa administration enhanced GABA efflux at primary motor cortex, which might be relate to partly the motor improvement, and sometimes the dyskinesia as well (Lindenbach et al., 2016). There are currently several types of GABA_A_ or GABA_B_ receptor positive modulator available, it will be interesting to understand which subtype of GABA receptor is involved in SST related cortical inhibition.

Apart from pharmacological tools, physical exercise improves motor behaviors efficiently in PD and other movement disorders. Exercise results in widespread, generalized changes in the brain, including enhanced secretion of many neurotrophic factors, restoration of neurotransmission, and increased stabilization of dendritic spines, partly through BDNF and mTOR signaling (Chen et al., 2019b). Cortical GABA dynamics is also associated with the beneficial effects of exercise training in physiological conditions, and rehabilitation processes (Coxon et al., 2018).

Another effective neuromodulation approach is the non-invasive transcranial magnetic stimulation (TMS). TMS pulses evoke cortical oscillation changes and dis-inhibit cortical dendrites by modulation cortical inhibition (Murphy et al., 2016). Repetitive TMS (rTMS) leads to enhanced cortical inhibition, which is mediated by increased GABA_B_R functioning. In fact, high frequency rTMS at primary motor cortex has demonstrated clinical efficacy in treatment PD patients, and improve motor rehabilitation in stroke patients (Vonloh et al., 2013). It is conceivable that enhanced cortical inhibition underlies the clinical benefits of rTMS on movement disorders.

Taken together, many lines of evidences co-suggest the importance of motor cortical inhibition in motor learning ability, and potentially other aspects of motor functioning. Targeting cortical inhibition with drugs, exercise, and TMS stimulation are feasible approaches for motor rehabilitation.

## Author Contributions

All authors designed the study together and wrote the manuscript together.

### Conflict of Interest

The authors declare that the research was conducted in the absence of any commercial or financial relationships that could be construed as a potential conflict of interest.
